# Risky Behavior: Hospital Transfers Associated with Early Mortality and Rates of Goals of Care Discussions

**DOI:** 10.5811/westjem.2020.5.46067

**Published:** 2020-07-08

**Authors:** Justin K. Brooten, Alyssa S. Buckenheimer, Joy K. Hallmark, Carl R. Grey, David M. Cline, Candace J. Breznau, Tyler S. McQueen, Zvi J. Harris, David Welsh, Jeff D. Williamson, Jennifer L. Gabbard

**Affiliations:** * Wake Forest School of Medicine, Department of Emergency Medicine, Winston-Salem, North Carolina; † Wake Forest School of Medicine, Department of Internal Medicine, Section on Gerontology & Geriatric Medicine, Winston-Salem, North Carolina; ‡ Wake Forest School of Medicine, Department of Internal Medicine, Section on General Internal Medicine, Winston-Salem, North Carolina; § University of North Carolina, Department of Emergency Medicine, Chapel Hill, North Carolina; ¶ Wake Forest Graduate School of Arts and Science, Department of Biomedical Science, Winston-Salem, North Carolina; || Wake Forest School of Medicine, Winston-Salem, North Carolina; # Wake Forest School of Medicine, Center for Health Care Innovation, Department of Internal Medicine, Winston-Salem, North Carolina

## Abstract

**Introduction:**

Inter-hospital transfer (IHT) patients have higher in-hospital mortality, higher healthcare costs, and worse outcomes compared to non-transferred patients. Goals of care (GoC) discussions prior to transfer are necessary in patients at high risk for decline to ensure that the intended outcome of transfer is goal concordant. However, the frequency of these discussions is not well understood. This study was intended to assess the prevalence of GoC discussions in IHT patients with early mortality, defined as death within 72 hours of transfer, and prevalence of primary diagnoses associated with in-hospital mortality.

**Methods:**

This was a retrospective study of IHT patients aged 18 and older who died within 72 hours of transfer to Wake Forest Baptist Medical Center between October 1, 2016-October 2018. Documentation of GoC discussions within the electronic health record (EHR) prior to transfer was the primary outcome. We also assessed charts for primary diagnosis associated with in-hospital mortality, code status changes prior to death, in-hospital healthcare interventions, and frequency of palliative care consults.

**Results:**

We included in this study a total of 298 patients, of whom only 10.1% had documented GoC discussion prior to transfer. Sepsis (29.9%), respiratory failure (28.2%), and cardiac arrest (27.5%) were the top three diagnoses associated with in-hospital mortality, and 73.2% of the patients transitioned to comfort measures prior to death. After transfer, 18.1% of patients had invasive procedures performed with 9.7% undergoing major surgery. Palliative care consultation occurred in only 4.4%.

**Conclusion:**

The majority (89.9%) of IHT patients with early mortality did not have GoC discussion documented within EHR prior to transfer, although most transitioned to comfort measures prior to their deaths, highlighting that additional work is needed in this area.

## INTRODUCTION

Adults with serious illnesses often visit an emergency department (ED) several times in their last year of life. Studies have shown that 75% of adults aged 65 and older with significant pre-existing conditions visit an ED within the last six months of life and 51% in the last month.^1^–^3^ Many of these patients receive aggressive and invasive intensive care interventions at the end of life, sometimes without clear benefit.^4^–^7^ This is especially true for patients subject to inter-hospital transfer (IHT) to a tertiary medical center, which occurs regularly and in up to 1.5% of all Medicare patients.^8^,^9^ Studies have shown that IHT patients have up to 2.7-fold increased risk of in-hospital mortality compared to non-transferred patients.^10^–^12^ In addition, up to 50% of these patients undergo inappropriate repeated procedures and tests.^13^ One study showed that IHTs was the most expensive non-therapeutic intervention performed in the acute setting.^14^

While a transfer may be necessary to ensure proper and timely care, transfers also can move patients to a location far from their families and may be associated with significant cost.^8^,^15^ In addition, studies have shown that IHT patients experience worse outcomes compared to non-transferred patients.^16^ Thus, given the increased cost, high mortality, and worse outcomes associated with IHT patients, having goals of care (GoC) discussions with patients and/or their loved ones prior to transfer is essential to ensure goal-concordant care.

Patients often choose less aggressive care if they anticipate a shorter life expectancy, lack of perceived benefit, and increased physical burden.^17^ One study demonstrated that aggressive end-of-life care just prior to death is later viewed as undesirable by bereaved families, compared to earlier transition toward comfort-focused measures.^18^ GoC discussions are associated with improved patient satisfaction, reduced healthcare costs, and reduced treatment burdens.^19^–^21^ However, there is a limited understanding of how often GoC discussions occur prior to transfer to a tertiary medical center. Our primary aim was to assess the prevalence of GoC discussions documented within the EHR and to assess the primary diagnosis associated with early mortality (defined as death within 72 hours of transfer) in patients who were transferred to a tertiary medical center.

## METHODS

### Study Design and Setting

This was a retrospective cohort study. We reviewed the EHR for all adults aged 18 years or older who had been transferred from an outside hospital to a tertiary medical center, Wake Forest Baptist Medical Center (WFBMC), between October 1, 2016–October 1, 2018 and expired within 72 hours of transfer. WFBMC is a Level 1 trauma center and serves the Piedmont Triad area of North Carolina, which is the north-central part of the state and contains 12 counties.^22^ The population is estimated at 1.69 million, making it the 30^th^ largest metropolitan area in the US. In the region, 22.2% of residents are African American and 15.9% are aged 65 and older.^22^ WFBMC is the only academic medical center in this 12-county region. This project was approved by the Wake Forest Institutional Review Board, with a waiver of requirement of informed consent.

**Population Health Research Capsule**

What do we already know about this issue?

*Patients transferred from community hospitals to tertiary medical centers are typically higher acuity, and at higher risk of mortality than non-transferred patients*.

What was the research question?

How often were goals of care (GoC) documented prior to transfer in patients who died within 72 hours of transfer?

What was the major finding of the study?

*GoC were documented prior to transfer in10% of cases, but was more likely in patients with a do-not-resuscitate order*.

How does this improve population health?

*Interhospital transfer can be a costly and potentially non-beneficial intervention. When possible, GoC should be explored prior to transfer*.

### Population

This study included all patients aged 18 and older who were transferred from any outside hospital to WFBMC between October 1, 2016–October 1, 2018, and expired within 72 hours of transfer. IHT patients under the age of 18 were excluded along with those who did not expire within 72 hours of transfer. A total of 298 patients met the inclusion criteria out of 16,506 admitted adult patients transferred from outside hospitals during the study period. One physician author verified the accuracy of the patient selection.

### Methods and Measurements

All data, with the exception of documented GoC discussions, primary diagnoses associated with mortality, and rates of palliative care consultation, were directly extracted from the EHR by a blinded data abstractor with training in biomedical informatics. We used Research Electronic Data Capture (REDCap) to record all study data.^23^ Demographic data collected included date of birth, age, gender, ethnicity, ZIP code, and marital status. The following information was also collected: transferring hospital name; date of admission; date of mortality; length of stay; primary diagnoses most contributing to death based on chart review; GoC discussion documentation in transfer medical records; utilization of palliative care consultation; use of mechanical ventilation; use of pressor agents; and code status prior to and after transfer.

GoC discussion documentation was obtained through manual chart review, and was defined as documentation of a discussion with the patient or surrogate decision maker(s) related to a “crisis communication.” This was further specified as any discussion about treatment decisions and goals related to what had brought the patient to the hospital.^3^,^24^ Each chart abstractor was instructed regarding documentation that would be considered as a documented GoC discussion, as just described. In cases of ambiguous documentation regarding GoC discussion, the chart was reviewed by a second chart abstractor to determine whether a GoC discussion was adequately documented to meet this description.

Manual chart review was performed to assess the primary diagnoses associated with in-hospital mortality. We pooled diagnosis categories to assess illness categories most associated with early mortality based on initial review of all encounter diagnosis codes. Contributing diagnosis determination was based on review of the admission and discharge notes, progress notes, and encounter diagnosis. In cases of ambiguous documentation, charts were reviewed by a second chart abstractor to determine primary and secondary contributing diagnosis for mortality. We calculated the Charlson Comorbidity Index (CCI) score based on hospitalization encounter diagnoses and patient problem list.^25^

Charts were manually reviewed to assess code status before and after transfer. If no documentation regarding pre-transfer code status was available, then we considered the pre-transfer code status to be full code as long as the initial documented code status was also full code after transfer. WFBMC currently has four tiers of scope of treatment orders: full code; do not resuscitate (DNR)-F (full scope of treatment); DNR-L (limited scope of treatment), and DNR-C (comfort care scope of treatment).^26^ There were two instances where documentation revealed patient/surrogate requests specifically for do-not intubate status. We compared pre-transfer and post-transfer code status for each patient to determine the frequency of change prior to death.

Rates of invasive procedures and major surgery after hospital transfer were also recorded based on manual chart review. We defined invasive procedures as diagnostic or therapeutic procedures other than mechanical ventilation or central line placement, as these were considered separately, and did not constitute major surgical procedures. Examples of invasive procedures include cardiac catheterization; cerebral angiography; direct intracranial pressure monitoring; inferior vena cava filter placement; mechanical thrombolysis; or tissue plasminogen activator administration. Major surgery was defined as any invasive operative procedure in which an extensive resection is performed (eg, a body cavity is entered, a partial or full organ is removed, or normal anatomy is altered). In-hospital cardiopulmonary resuscitation (CPR) rates after transfer and rates of palliative care consultation were also recorded based on manual chart review. The frequency of palliative care consultation was manually assessed through review of transfer documentation, consultation orders, and progress notes.

### Statistical Methods

We used descriptive statistics of means and standard deviations (SD) for panel demographic and encounter data. Microsoft Excel, version 16.0 (Microsoft Corporation, Redmond, WA) was used for all analyses; p<0.05 was assumed to be significant. We used chi-squared test to compare proportion of GoC discussions between institutions, and from referral ED or referral inpatient settings, as well as correlation between frequency of documented GoC discussions and code status prior to transfer. Chi-squared test was also used to assess aggregated code status outcomes following transfer. We used test of proportion to compare percentage of males to females in patient cohort, as well as prevalence of change in code status before and after transfer.

## RESULTS

### Patient Demographics

A total of 298 patients were transferred from inpatient settings and EDs at 51 outside community hospitals to WFBMC between October 1, 2016–October 1, 2018 and expired within 72 hours of transfer. The majority (57.7%) of patients were aged 65 or older, with 53% being male (Table 1). The median unadjusted CCI score for patients 18 and older to less than 65 years of age was 1 (SD 2.4), while the median unadjusted CCI for patients age 65 and older was 3 (SD 2.1).

### Goals of Care Documentation

GoC discussions were documented in 10.1% (n = 30) of patients prior to transfer to the tertiary medical center. In those patients transferred directly from the ED 8.5% (n = 19) had GOC documentation, and in those transferred directly from inpatient settings, including floor and intensive care unit, 14.7% (n = 11) had documented GoC discussions. There was no significant difference (p = 0.12) between the frequency of documented GoC discussions prior to transfer for patients coming directly from the ED vs inpatient settings (Table 2).

### Primary Diagnoses Associated with In-Hospital Mortality

The median length of stay was 32.9 (SD 19.46) hours with 41.9% and 72.5% of patients dying within 24 and 48 hours after transfer, respectively. Sepsis (29.9%), respiratory failure (28.2%), and cardiac arrest (27.5%) were the top three primary diagnoses associated with in-hospital mortality (Figure). Cardiac arrest was only included as a contributing diagnosis to the patient’s transfer mortality for patients who were transferred to the hospital following return of spontaneous circulation from a pre-transfer cardiac arrest event, and not solely as a terminal event of tertiary hospitalization. Notably, hemorrhagic stroke was the most specific diagnostic category after sepsis, respiratory failure, and cardiac arrest, affecting 10.4% of patients.

### Code Status and Scope of Treatment Changes

The majority (90.3%) of patients were full code prior to transfer, and in 85.9% (N = 231) their code status was changed to DNR within 72 hours of transfer. In 73.2% (N = 218) of patients, their status was transitioned to comfort measures prior to death (Table 3). Thirty percent (N = 89) of patients underwent in-hospital CPR after transfer. Of the patients (N = 29) who were not full code prior to transfer, 72.4% (N = 21) were further de-escalated to comfort measures (DNR-C) prior to death. Only one patient had escalated care after transfer from DNR-F initially to full code prior to death.

Patients with a DNR prior to transfer were more likely to have a documented GoC discussion prior to transfer compared with patients who were full code prior to transfer (Table 4).

There was no significant difference between groups with aggregated code status outcomes following transfer. The data suggested a trend towards higher prevalence for comfort care among patients who had documented discussions prior to transfer, but this was not significant (Table 5).

### Invasive Procedures, Surgery, and Palliative Care Consultation Rates

A total of 18.1% (N = 54) of patients underwent invasive procedures, and 9.7% (N = 29) underwent major surgery prior to death. Palliative care was consulted for only 3.4% (N = 10) of patients after transfer and 1.0% (N = 3) of patients prior to transfer.

## DISCUSSION

This study revealed that only 10.1% of patients had documented GoC discussions prior to transfer, although the majority (73.2%) of patients had a de-escalation of code status within 72 hours of transfer to comfort measures. GoC discussions are critical, allowing patients and their families to be well informed of proposed therapies along with their risks and benefits. GoC discussions are associated with improved patient satisfaction, reduced healthcare costs, and reduced treatment burdens.^19^–^21^ Patients commonly present to an ED because there has been an acute change in their overall health, often representing an inflection point in their trajectory of illness. Emergency physicians are called upon to conduct initial GoC discussions particular to that crisis situation.^3^,^27^,^28^ Shared decision-making can only occur after GoC discussions have examined the patient’s preferences and values.^3^,^29^ Unfortunately, our study highlighted that these discussions occur infrequently, resulting in patients possibly receiving unwanted and unnecessary aggressive care in the last days of life. Further research is needed to investigate the best strategies for training emergency physicians in conducting GoC discussions and implementing standardized ways to document these discussions within the EHR.

There was a predominance of mortality associated with sepsis, respiratory failure, post-cardiac arrest care, and acute neurologic conditions including hemorrhagic stroke in this cohort. These patients suffered early in-hospital mortality despite receiving aggressive care interventions, including IHT, possibly due to lack of early predictors for poor prognostic outcome. It is also possible that recognition of patients at a high risk of mortality despite transfer was missed by transferring physicians and that further training in this area is needed.

Research has shown that early palliative care consults in the ED can decrease hospitalization cost, in-hospital mortality, and length of stay while improving quality of care and availability of acute bereavement support for families.^30^–^32^ Unfortunately, only 4.4% of patients in this study had a palliative care consult. These results are not dissimilar to other studies showing that emergency physicians are less likely to refer patients to palliative care, representing only 3% of palliative care referrals.^29^ Given the severity of disease and poor prognosis in this patient cohort, we expected a higher consultation rate.^33^–^38^ It is possible that providers did not consider involvement of palliative care until all life-prolonging measures had been attempted or exhausted. This is supported by the low rates of documented GoC discussions, leaving little time to involve palliative care providers prior to a patient’s death. The high mortality rate associated with IHT, especially in those with sepsis, respiratory failure, recent cardiac arrest, and neurologic emergencies, highlights the need for early GoC discussions with these patients to ensure quality and goal-concordant end-of-life care.

While transfers to tertiary care centers are ostensibly pursued to give patients access to resources, treatments, or procedures that are not available at the referring institution, we were surprised to see the relatively small portion of patients in this study who underwent surgeries or invasive procedures (9.7% and 18.1%, respectively). We are not aware of similar prior studies comparing rates of GoC discussions in patients with early mortality with rates of intervention following transfer. Multiple inferences can be made regarding the low rates of invasive or surgical procedures. The majority of patients had diagnoses that were medical in nature, but presumably even patients with medical diagnoses were transferred with the potential for specific invasive interventions. It is also possible that due to disease progression throughout the transfer period, many patients were poor candidates for these therapies at presentation to our tertiary care center.

Disease progression may have also impacted the GoC for the patient or family, rendering these invasive therapies no longer goal concordant. Further study is needed to differentiate the factors contributing to differences in pre-transfer assessment of the need for a higher level of care with subsequent care or interventions offered following transfer. Given the expanding availability of detailed medical record exchange through EHR networks, specialist evaluation of candidacy for invasive procedures prior to transfer may become standard practice in the future.

The vast majority of patients (84.6%) had a change in their code status and scope of treatment after transfer with 73.2% of patients receiving comfort measures only prior to death. We suspect that this was multifactorial. Some patients may have been deemed poor candidates for aggressive therapies upon transfer or additional diagnostic information may have been available to providers leading to more accurate prognostication that was communicated to patients and their surrogate decision makers. More study is needed to determine the specific aspects of communication or prognostication that may have influenced decision-making following transfer, all of which can be documented in varying detail by medical providers. Standardized documentation of code status changes, preferences regarding care goals, stipulations of management, quality of life considerations, and other aspects of care in the EHR can help address dynamic changes in condition and goals that may occur during hospitalization.^39^

## LIMITATIONS

There are several potential limitations in our study. This was a single-center study, which may affect the generalizability of the results; and external validity is lacking. We assessed rates of GoC discussions based on documentation within the EHR, which likely has high inter-provider variability, particularly given the busy nature of the ED. The different transferring institutions use multiple EHR systems that may not communicate with the receiving hospital; thus, review of GoC discussions included review of available records provided at the time of transfer, which may not have been complete. The rates of these discussions could have been higher although just not documented in EHR.

The retrospective nature of this study can result in potentially ambiguous baseline data. Also, based on chart review, we suspect that the CCI of this population was not adequately captured. This is possibly due to a lack of thorough history of patient comorbidities given acuity of presenting condition, inaccurate recording of significant comorbidities in a patient’s problem list, patients being too ill to adequately relay their history to providers, and patients being transferred by outside hospitals and health systems which may not have EHR systems capable of communicating with the receiving institution’s EHR. Additionally, any informal guidance provided by palliative care providers via phone or after regular consultation hours would not be captured in review since consultation was only considered if there was a consult order placed during hospitalization or direct documentation of consultation by a palliative care provider.

We assessed whether or not a GoC discussion took place, based on minimum specific criteria, but the depth or utility of such discussions may vary widely among medical providers. The chart abstractors were not blinded to study hypothesis. While a secondary review of charted GoC conversations and contributing diagnoses was undertaken in cases of ambiguous documentation, we did not perform review of each chart by a second reviewer to evaluate inter-rater reliability.

## CONCLUSION

Goals of care discussions were infrequent in this cohort of IHT patients. Based on prior research on the outcomes of IHT patients and the effects of GoC discussions we suspect that early delivery of prognostic information and GoC discussions may have prevented some of these transfers from occurring, thereby possibly improving patient and family satisfaction, reducing treatment burden, and reducing costs.^16^–^21^ The majority of patients in this study came from ED settings. Barriers to GoC discussions occurring in ED settings likely include time limitations, provider comfort level with these discussions, lack of training in conducting GoC discussions, and availability of palliative care resources for potential care transitions.

Further study is needed to better understand the complexity of this issue and potential solutions. Based on this data, we suspect that facilitating early involvement of palliative care in patients at high risk of mortality prior to transfer could help identify patients who may not benefit from or want an inter-hospital transfer. In settings that lack direct access to a palliative care provider, targeted education for providers as well as telemedicine-based palliative care support may help bridge this gap.

## Figures and Tables

**Figure:**
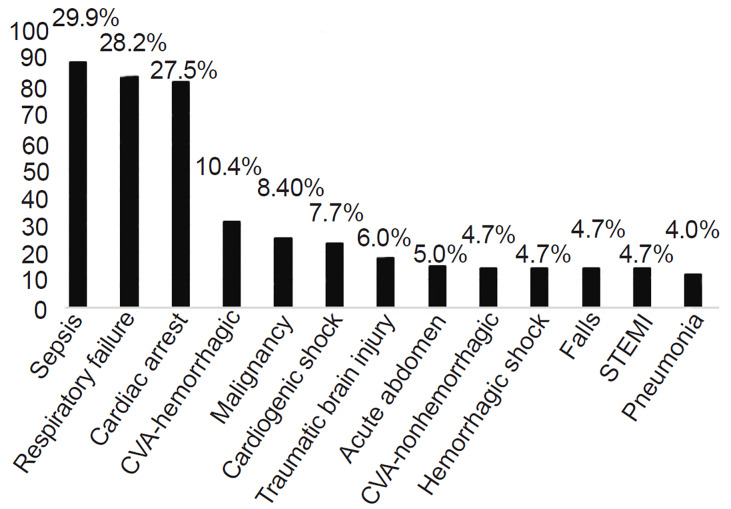
Primary diagnoses associated with in-hospital mortality. *CVA*, cerbrovascular accident; *STEMI*, ST-elevation myocardial infarction.

**Table 1:** Baseline demographic data of study cohort of patients transferred to a tertiary care hospital.

Variable	N (%)
Age	
18 to <65	126 (42.3)
≥ 65	172 (57.7)
Gender	
Male	158 (53.0)
Female	140 (47.0)
Race/Ethnicity	
White	260 (87.2)
African-American	26 (8.7)
Hispanic or Latino	5 (1.7)
American Indian or Alaskan Native	3 (1.0)
Other/unknown	4 (1.3)
Marital status	
Single, never married	67 (22.5)
Married	150 (50.3)
Divorced	32 (10.7)
Widowed	46 (15.4)
Separated	3 (1.0)
Comorbidities	
Diabetes mellitus	88 (29.5)
Liver disease	30 (10.0)
Cancer	76 (25.5)
HIV/AIDs	1 (0.3)
Chronic renal disease	47 (15.8)
Congestive heart failure	66 (22.1)
Coronary artery disease	45 (15.1)
COPD	69 (23.1)
Peripheral vascular disease	15 (5.0)
Cerebrovascular accident/TIA	54 (18.1)
Dementia	15 (5.0)

*HIV*, human immunodeficiency virus; *AIDS*, acquired immunodeficiency syndrome; *COPD*, chronic obstructive pulmonary disease; *TIA*, transient ischemic attack.

**Table 2:** Frequency of goals of care discussions prior to transfer from emergency department and inpatient settings.

Pre-transfer location	Patients per setting N (%)	GOC discussions by setting N (%)	P-value
Emergency department	223 (74.8%)	19 (8.5%)	p= 0.12
Inpatient settings	75 (25.2%)	11 (14.7%)	
Total GoC discussions		30 (10.1%)	

*GoC*, goals of care.

**Table 3:** Code status before transfer and after transfer prior to death.

Code Status	Before transfer N (%)	After transfer N (%)	P-value
Full Code	269 (90.3%)	38 (12.8%)	p < 0.00001
DNR Status	29 (9.7%)	258 (86.6%)	p < 0.00001
DNR/Full SOTO	14 (4.7%)	22 (7.4%)	p = 0.168
DNR/Limited SOTO	15 (5.0%)	18 (6.0%)	p = 0.589
DNR/Comfort Care SOTO	0 (0.0%)	218 (73.2%)	p < 0.00001
Do Not Intubate	0 (0.0%)	2 (0.7%)	p = 0.156

*DNR*, do not resuscitate; *SOTO*, scope of treatment order.

**Table 4:** Frequency of goals of care discussions prior to transfer compared to code status prior to transfer.

GOC Documentation Prior to Transfer	DNR Prior to Transfer N (%)	Full Code Prior to Transfer N (%)	P-value
Documented GoC Discussion	8 (26.7%)	22 (73.3%)	p = 0.002
No Documented GoC Discussion	20 (7.5%)	248 (92.5%)	

*GoC*, goals of care discussion; *DNR*, do not resuscitate.

**Table 5:** Code status after transfer compared to documentation of goals of care discussion prior to transfer.

GOC Documentation Prior to Transfer	Full Code	DNR-F N (%)	DNR-L N (%)	DNR-C N (%)	P-value
Documented GoC Discussion	3 (10.0%)	1 (3.3%)	1 (3.3%)	25 (83.3%)	p = 0.591
No Documented GoC Discussion	37 (13.8%)	21 (7.8%)	17 (10.1%)	193 (72.0%)	

*GoC*, goals of care; *DNR-F*, do not resuscitate-full scope of treatment; *DNR-L*, DNR-limited scope of treatment; *DNR-C*, DNR-comfort scope of treatment.
